# Monthly Summary

**Published:** 1871-03

**Authors:** 


### MONTHLY SUMMARY.

*Styptic Wool.*—Dr. Ehrle, of Isny, in the interest of his countrymen wounded in the present war, desires to make known a very simple preparation of wool that he has found very serviceable in arresting hæmorrhage after operations or from wounds. To prepare it he boils the finest carded wool for half an hour or an hour in a solution containing four per cent. of soda, then thoroughly washes it out in cool spring water, wrings and dries it. The wool is thus effectually purified, and is now capable of imbibing fluids uniformly. It is then to be dipped two or three times in fluid chloride of iron diluted with one-third of water, expressed and dried in a draught of air, but not in the sun or with high heat; finally it is carded out. Thus prepared, it is of a beautiful yellow color, and feels like ordinary dry cotton wool. As it is highly hygroscopic, it must be kept dry, and when required to be transported must be packed in caoutchouc or bladder. Charpie may be prepared in a similar manner, but on account of its coarse texture, is not so effective as cotton wool, presenting a less surface for coagulation. When the wool is placed on a bleeding wound, it induces moderate contraction of the tissue, coagulation of the blood that has escaped, and subsequently coagulation of the blood that is contained within the the injured vessels, and thus arrests the hæmorrhage. The coagulating power of the chloride of iron is clearly exalted by the extension of its surface that is in this way effected. The application of the prepared wool is not particularly painful, whilst, by sucking up the superfluous discharge and preventing its decomposition, it seems to operate favorably on the progress of the wound. The unpleasant secondary results that have led many practical surgeons to discard the use of the pechloride of iron do not occur with the wool when it is properly made and applied. In cases of wounds where the bleeding proceeds from large and deep seated vessels, it may be used as a compress, a bandage being applied over it, or the wound may be plugged with it. It may also be employed with advantage in cases of profuse suppuration, to imbibe the discharge and purify the surface. He recommends that a small portion should be given to every soldier on going into action.—*Lancet.*

*Dangers of Snuff-Taking.*—In one of our hospital reports will be found a startling instance of the poisonous effects of lead being produced by the use of contaminated snuff. The victim who was a resident in India, was in the habit of taking "best brown rappee," exported by a well known and popular English firm in leaden cases. His medical advisers first attributed the failing power of his upper extremities, and other unpleasant symptoms, to the use of his favorite tobacco; but, after he had been to the trouble and expense of coming to England in search of further advice, his suffering was traced to the real source. To be quite certain, six separate packages were ordered from Calcutta, and a sample from each of them was found to contain an intimate union with a considerable quantity of lead. Rappee appears to be a rather moist preparation of the refreshing weed, and, where it adhered to the sides of the leaden cases, it was in these instances found to be dotted with spots of carbonate of lead, formed, it is supposed, by a combination with the walls of the case, of carbonic acid, liberated by the fermentation of the damp snuff: it presented, in fact, a miniature example of the process by which white lead is manufactured for commercial purposes. A medical man, who has recently returned from Calcutta, states that he had lately met with three patients suffering in the same way from the same cause. They are probably not the only snuff-takers who are being poisoned with lead. Both manufacturers and consumers would do well to see to it at once that damp provisions are no longer stored in leaden cases.—*Lancet.*

*** The above remarks suggest an inquiry whether the common practice of putting up chewing tobacco in lead foil may not account for some of the numerous cases of lead poisoning not traced to any recognized source. Ed. M. G.—*Med. Gazette.*

---

*Simple Method of Arresting Obstinate Epistaxis.*—A writer in the *Gazette des Hopitaux* states that attentive observations of the face of a patient attacked with epistaxis will detect a slight intermittent movement of the soft parts near the ala of the nose on the side where the blood is flowing; even if this pulsation be not seen, it may be felt. Pressure with the finger over this branch of the facial artery is said to arrest the hemorrhage immediately.—*Med. Gazette.*

*Borax.*—It may not be generally known how very valuable borax is in various purposes of household use. We find it the very best cockroach exterminator yet discovered. One half-pound, costing but fifty cents, has completely cleared a large house formerly swarming with them, so that the appearance of one in a month is quite a novelty. The various exterminating powders puffed and advertised have been found not fully effective, tending rather to make the roaches crazy than to kill them. There is something peculiar, either in the smell or touch of borax, which is certain death to them. They will flee in terror from it, and never appear again where it has once been placed. It is also a great advantage that borax is perfectly harmless to human beings; hence no danger from poisoning. It is also valuable for laundry purposes. The washerwoman of Holland and Belgium, so proverbially clean, and get their linens so beautifully white, use refined borax as washing powder instead of soda, in the proportion of a large handful of borax powder to ten gallons of water. They save soap nearly one-half. All the large washing establishments adopt the same mode. For laces, cambrics, etc., an extra quantity of the powder is used; and for crinolines (requiring to be made stiff) a stronger solution is necessary. Borax, being a neutral salt, does not in the slightest degree injure the texture of linen. Its effect is to soften the hardest water, and therefore it should be kept on the toilet table. As a way of cleaning the hair, nothing is better than a solution of borax in water.—*Manufacturer and Builder.*

---

*Treatment of Wounds by Pneumatic Occlusion.*—One of the last numbers of the *Gazette Medicale de Paris* contains a suggestion by M. Guerin for treating wounds by the exclusion of the atmosphere. This is accomplished by placing over the wound a small sac or cylinder of caoutchouc, to the extremity of which a pipe is inserted that is attached to a balloon or chest destitute of air, and which consequently exerts a constant suction power over the seat of injury. By this means, according to M. Guerin, the approximation of the edges of the wound, and their consequent healing by first intention, is facilitated, while it prevents the absorption of pus. By this means, in fact, the wound is placed in the condition of a subcutaneous injury.—*Lancet.*

*Death from Chloral Hydrate.*—Dr. George Needham reports in the *Journal of Psychological Medicine* a case of fatal cerebral congestion following the administration of Hydrate of Chloral to a married woman, aged 50, of hysterical diathesis, who had suffered for some two years with symptoms of mental derangement, consisting of distressing "nervousness," fear of impending death, hesitation, suspiciousness, etc. Ophthalmoscopic examination showed an enlarged and tortuous condition of the retinal vessels. In October, 1870, the loss of a relative threw her into a state of much excitement, for which she took, on October 19th, 115 grains of bromide of potassium. On the 21st, chloral hydrate was prescribed in thirty-grain doses, of which she took six, as follows:—On the 21st, at 5 30 p.m. and 11 p.m.; on the 22d, at 10 a.m. and 3 p.m.; on the 23d, at 1 a.m., 8.10 a.m., and 1.30 p.m. On the afternoon of the 22d she was sleeping quietly, with a somewhat rapid pulse, and was found in the same condition at two visits (morning and evening) on the 23d. On the morning of the 24th, her continued sleep created alarm, and ineffectual attempts were made to rouse her, which were maintained during the day and night. Sulphate of strychnia was thrice injected in doses of one thirtieth of a grain at intervals of four hours during the night. Coma progressed to a fatal termination on the afternoon of the 25th. The autopsy revealed extreme hyperæmia of the pia mater and brain substance. A year before, the patient had taken nearly the same quantity of chloral within the same period of time without ill effects. The writer suggests that the previous administration of a long course of bromide of potassium may increase the danger of full doses of chloral.—*Med. Gazette.*

---

*Adulterations of Vinegar.*—It is a well known fact that few articles of commerce are more adulterated than vinegar. Cayenne pepper and some bitter root are used, but oftener less harmless and even poisonous substances are mixed with the vinegar.

A very simple means of detecting these adulterations is the following:—Neutralize the vinegar with carbonate of soda, concentrate the obtained solution by boiling it down to about one-half its quantity and let it cool. If it leaves a hot and burning taste on the tongue, it is adulterated.—*Med. Gazette.*

*Health*—In a recent address, Lord Stanley said to some men starting in life:—

"Let them recollect that the race in which they are engaged is one which will last all their working days, and in which endurance is more important than speed. They know already that intemperance is one form of suicide—let them remember that all neglect of physical laws is open to the same reproach, and that there may be intemperance in work as well as in meaner indulgences. You cannot have mental efficiency without health; you cannot maintain health in an exciting and exhausting employment, such as most mental occupations are, when eagerly followed, without some care to preserve it. What I had to tell you about keeping up literary tastes has been said already. They are a great pleasure—a wholesome and a lasting one. Keep them up if you can, and as long as you can. But while you do that, remember also that there is one thing as compared with which not only material health and luxury, but all art, all literature, all science even, are relatively unimportant, and in regard to which the poorest laborer may compete with any of you on equal terms—I mean a steady, untiring performance by each individual of his public and private duty."

---

*To Detect Manufactured Rum or Brandy.*—Mix the liquid to be tested with about one-third its quantity of concentrated English sulphuric acid of 1.840 sp. gr. If pure, there remains, after cooling, the specific aroma or flavor of the liquid, which is not lost even after 24 hours; whereas, if adulterated, the flavor is lost. The sulphuric acid itself or the heat it evolves seems to destroy the essential oils used for flavoring.—*Med. Gazette.*

---

*Death from the Inhalation of Ether.*—In the number of the *Boston Medical and Surgical Journal* for Dec. 8, 1870, there is reported by Walter Burnham, of Lowell, a case of "death from the effects of sulphuric ether," in the opinion of the editor "the result of an overdose." The operation was an amputation of the thigh for a gunshot wound; an ounce of ether was administered on a small napkin in a bowl, which was placed over the face, and one or two drachms were added every two or three minutes. In about ten minutes anæsthesia was induced, and the ether suspended; slight sensibility returning the ether was renewed with the desired effect, and again suspended. The operating surgeon then ordered the napkin to be reapplied with a drachm of ether freshly poured upon it. After one or two inspirations the patient ceased to breathe.—*Med. News.*

---

*The Career of Dentists.*—A dentist in Philadelphia has traced out the career of 1,000 dentists, with this result:—163 died before they reached middle life, 643 attained fair success, 57 made fortunes, 27 died from intemperance and other vices, 96 failed entirely, and 3 committed suicide.—*Boston Medical and Surgical Journal.*

---

*Modern Surgery.*—An illustration of the advance in surgery made in the present century may be found in the fact that the operator who first successfully tied the internal iliac artery for aneurism, Dr. Stevens, died only two years ago. Dr. S., who then lived in the West Indies, was only twenty-six years of age, in 1812, when he achieved this feat. Nine other operations of this kind, performed since, are upon record, with four successes, so that the proportion of deaths is only 50 per cent.—*Richmond and Louisville Medical Journal.*

---

*Asthma.*—In an interesting discussion on the management of asthma, by the members of the *Baltimore Pathological Society*, Dr. Garretson, (*Baltimore Medical Journal*) alluded to the case of Colonel Washington, subject to attacks of this affection lasting four or five days, who was relieved in two days by bromide of potassium and belladonna.

---

*Skin-Grafting Superseded.*—Recent statements on the interesting subject of skin-grafting seem to show that they were mistaken who at first thought it necessary to transplant pieces of skin of any depth. All observers now agree that the first phenomenon is the disappearance of the characters of true skin in the piece transplanted, which becomes a mere pelicle. Further, there should be no trace of areolar or adipose tissue left, and Dr. Fiddes says that there is no necessity to take any skin, but merely a few eperdermic scales.—*Med. Press and Circular.*

*The Seat of Tumor.*—Virchow (*N. Y. Med. Journal*) states that those organs which possess a soft surface, and are often brought in contact with foreign bodies, are oftener the seat of tumors than those which are shut up in cavities, and are rarely brought in contact with outside influences.

---

*Carbolic Acid in Carbuncle.*—Dr. J. C. Nott, of New York [*N. Y. Med. Journal*], advocates the local use of carbolic acid in carbuncle, and publishes an obstinate case which was cured after seven applications. He believes this acid to be more penetrating in its action, more efficient as an antiseptic, than subsulphate of iron.—*Med Record.*

---

